# Gestational Gigantomastia Complicating Pregnancy: A Case Report and Review of the Literature

**DOI:** 10.1155/2015/892369

**Published:** 2015-12-02

**Authors:** Shadi Rezai, Jenna T. Nakagawa, John Tedesco, Annika Chadee, Sri Gottimukkala, Ray Mercado, Cassandra E. Henderson

**Affiliations:** ^1^Department of Obstetrics and Gynecology, Lincoln Medical and Mental Health Center, Bronx, NY 10451, USA; ^2^School of Medicine, St. George's University, West Indies, Grenada

## Abstract

*Background*. Gestational gigantomastia is a rare disorder without clear etiology or well-established risk factors. Several pathogenic mechanisms contributing to the disease process have been proposed, all of which can lead to a similar phenotype of breast hypertrophy.* Case*. A 28-year-old Guinean woman presented at 37 weeks of gestation with bilateral gigantomastia, mastalgia, peau d'orange, and back pain. Prolactin levels were 103.3 *μ*g/L (with a normal reference value for prolactin in pregnancy being 36–372 *μ*g/L). The patient was treated with bromocriptine (2.5 mg twice daily), scheduled for a repeat cesarean, and referred to surgery for bilateral mammoplasty.* Conclusion*. Gestational gigantomastia is a rare disorder, characterized by enlargement and hypertrophy of breast tissue. Our patient presented with no endocrine or hematological abnormalities, adding to a review of the literature for differential diagnoses, workup, and management of cases of gestational gigantomastia with normal hormone levels.

## 1. Introduction

Gigantomastia in pregnancy, also known as gestational gigantomastia (GG) and gravid macromastia, is a rare condition that presents as breast enlargement during pregnancy [1, 2]. The first case of GG was described by Palmuth in 1648 [3–5]. Since his initial description, approximately 100 cases have been reported in the literature [4, 6, 7]. The incidence of GG has been reported to be between 1 in 28,000 and 1 in 100,000 pregnancies between the years of 1935–1960 [2, 6] and 1989–2009, respectively [8–10]. Gigantomastia usually affects both breasts, but it may occur unilaterally and, in some cases, may persist beyond pregnancy [9, 11–13].

The term gestational gigantomastia or macromastia [14] refers to breast enlargement during pregnancy while that occurring during puberty is called “pubertal macromastia.” In addition, macromastia (gigantomastia) classically is referred to as “massive enlargement of breasts in non-obese women in whom weight bearing itself is uncomfortable and stretching of overlying skin causes ulceration.” In 2011, Dafydd et al. defined gigantomastia as excess breast tissue that contributes >3% of a patient's total body weight [15].

## 2. Presentation of Case

**Figure 1:**
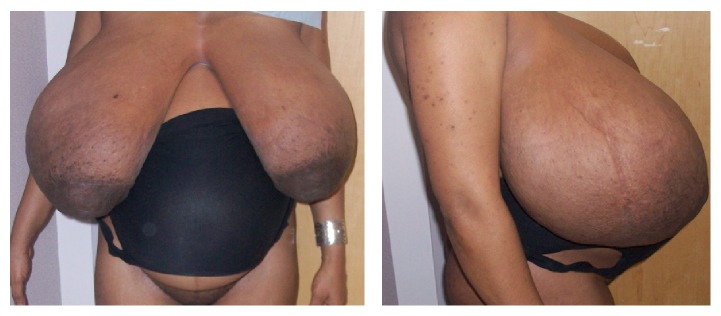
Patient with gestational gigantomastia at 37 weeks (initial presentation).

**Figure 2:**
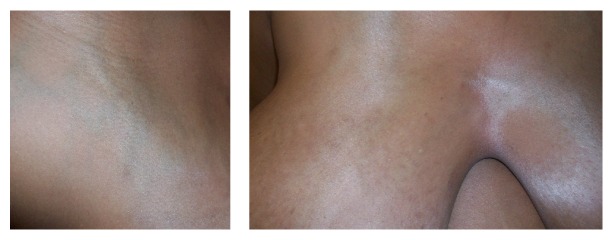
Dilated veins on the upper thorax in a patient presenting at 37 weeks with gestational gigantomastia.

**Figure 3:**
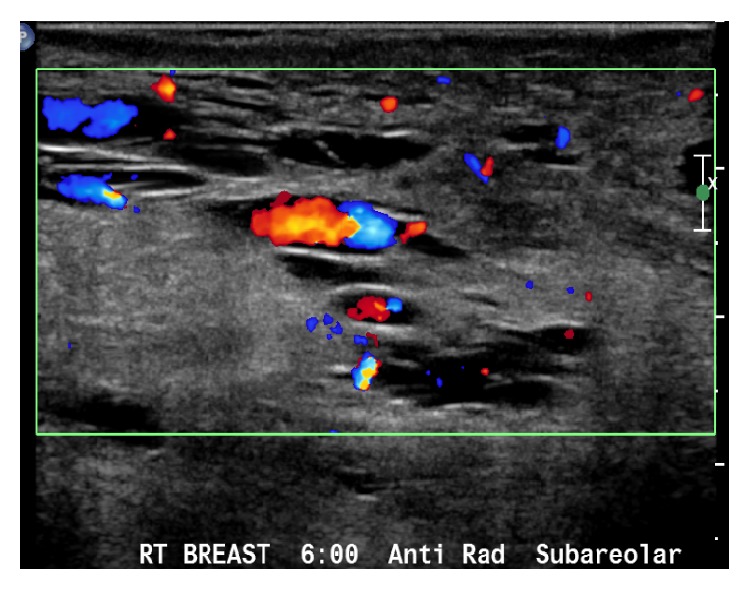
Breast ultrasound: 6 o'clock: huge ducts that should be going in the direction of the nipples (but there are not!). These dilated ducts end bluntly and are not connected to the outside. Therefore, the fluids and materials in them cannot decongest and stay as fluid filled.

In July 2014, a 28-year-old G2P0101 Guinean woman presented for her first prenatal visit at 37 weeks of gestation with bilateral gigantomastia (Figure 1). The patient reported severe back pain, dyspnea, and marked breast tenderness. During the initial interview, the patient reported that breast enlargement started at 3 months of gestation. She reported that this was the first time she had experienced excessive breast enlargement. This had not occurred in her previous pregnancy. Past medical history was unremarkable. Past surgical history included only a cesarean at 36 weeks in New Guinea (Africa) for worsening preeclampsia. She reported that her breast size prior to pregnancy was 34B and estimates her breasts are now 5 times bigger during this pregnancy. She is now wearing a size 44-DD brassiere which is very tight/small and does not fit her breasts. Physical exam was remarkable for distended and indurated superficial veins on the upper thorax (Figure 2) and altered spinal curvature. Measurements on the right breast were circumference 75 cm, from axilla to nipple 45 cm, and nipple to midline 37 cm (Table 1). Her breast size remained at these dimensions for the duration of the pregnancy. Serum prolactin measurements included an initial level of 103.3 *μ*g/L (with a normal reference value for prolactin in pregnancy being 36–372 *μ*g/L) [16] and a postpartum day 6 level of 40.4 *μ*g/L (up to 100 *μ*g/L is normal [17]). Other laboratory investigations—white blood cell count, hemoglobin, hematocrit, platelet count, basic metabolic panel, alkaline phosphatase, and coagulation studies—remained within normal limits throughout her pregnancy. The patient was treated with bromocriptine 2.5 mg twice daily and referred to radiology for breast sonography. Ultrasound modality demonstrated skin thickening and linear hypoechoic areas (due to dilated ducts and vascular structures) (Figure 3), with no abnormal growth—findings consistent with breast hypertrophy. During the initial evaluation, breast reduction was not recommended due to potential surgical complications. The patient declined trial of labor after cesarean. Therefore, an elective repeat cesarean delivery was performed at 39 weeks with delivery of a 3160 g female with Apgar scores of 7 (1 min) and 8 (5 min).

Due to persistent neck, shoulder, and back pain, the patient initially elected to stop breastfeeding and to continue bromocriptine treatment (2.5 mg twice daily); however subsequently patient changed her mind and opted for breastfeeding and cessation of bromocriptine. She was evaluated by plastic surgery at 3 weeks and 6 weeks postpartum for a bilateral breast reduction. Upon reevaluation at 6 weeks postpartum, the patient had no active complaints; she decided to postpone the breast reduction surgery as she was breastfeeding her baby. The patient returned to New Guinea in Africa. At 8 months postpartum the patient had a bilateral breast reduction surgery in Belgium.

## 3. Discussion

Risk factors for gestational gigantomastia are not well understood, but occurrence is more common in Caucasian and multiparous women [18]. Gestational gigantomastia can occur during any pregnancy [19]. A prior history of GG increased the risk for the condition in subsequent pregnancies [7, 9, 12, 20]. Furthermore, rates of recurrence have historically been increased in patients who underwent reduction mammoplasty instead of bilateral total mastectomy, the recurrence being attributed to retained hypertrophic tissue after mammoplasty [3, 7, 21].

The etiology and pathogenesis of gestational gigantomastia remain elusive [22], but many theories have been proposed, including excessive production of estrogen or prolactin, hormone receptor sensitivity, and underlying autoimmune disease triggered by pregnancy [14]. There are also reported cases of penicillamine induced breast gigantism [23–26] with subsequent treatment with danazol [25, 26].

Since most cases of gigantomastia occur during puberty or pregnancy, one possible etiology is the excessive release of estrogen or prolactin [5, 27, 28]. However, many cases of gigantomastia have occurred in a setting of normal hormone levels or even after medical suppression with bromocriptine [29]. Even in a setting with high estrogen and prolactin levels, it is unclear whether such levels, normally elevated in pregnancy, are pathogenic [13, 30].

Cases of gigantomastia with normal hormone levels may be explained by increased hormonal sensitivity in the target organ [2, 27, 31]. In 2002, Agarwal et al. [31] presented a case of a 24-year-old gravida 2, presenting at 19 weeks of gestation with bilateral breast hypertrophy. Prolactin levels and all other lab investigations were normal. The authors suggested that estrogen receptor sensitivity to prolactin might have accounted for breast hypertrophy and enlargement [31]. Increased hormone receptor sensitivity may also account for cases of unilateral gigantomastia [13]. However, this theory of hormone receptor hypersensitivity does not account for primary presentation in multigravida women [32].

In addition to aberrant hormone levels or possible hormone receptor sensitivity, several authors have suggested a possible autoimmune etiology to gigantomastia [33–35]. Several cases of gigantomastia have been described in association with autoimmune disorders, such as systemic lupus erythematosus (SLE), myasthenia gravis, Graves' disease, and rheumatoid arthritis [18, 19, 23, 36]. Vinicki et al. proposed that pregnancy could be a trigger for SLE emergence [37], suggesting that gigantomastia in pregnancy might be the result of an underlying autoimmune disorder.

Physical complications seen in gigantomastia include rapid breast enlargement leading to severe pain and tenderness, ulcerations, necrosis, and hemorrhage [20, 29, 38, 39]. Secondary infection (e.g., puerperal mastitis, pyogenic abscess) and sepsis may also occur in the absence of appropriate medical treatment [6, 19, 20, 39, 40]. Apart from physical complications, patients are often ostracized due to their physical appearance and suffer psychological trauma, depression, and sociophobia [3, 5–8, 10].

Before making a diagnosis of benign gestational gigantomastia, other disease processes must be considered. Differential diagnoses for GG with normal prolactin levels may include infectious mastitis, juvenile breast hypertrophy and/or normal pregnancy changes, benign breast conditions such as fibrocystic change or fibroadenoma, and underlying malignancy. In addition to rapidly enlarging breasts, patients with fever, localized tenderness, and erythema of skin overlying the breasts may receive an initial diagnosis of mastitis [41]. Histologic findings of normal breast tissue have led some researchers to suspect juvenile breast hypertrophy in young pregnant women, or normal pregnancy-related breast enlargement, especially in early stages when breast growth was within physiologic limits [42]. Among women with histories of fibrocystic change or fibroadenomas and histologic findings consistent with the aforementioned histories, researchers have suspected recurrence of these benign breast disorders before reaching a diagnosis of gigantomastia in pregnancy [2, 32, 43].

Because of the rapid rate of breast enlargement, GG presents similarly to malignant breast disorders such as a phyllodes tumor [7, 12]. Other cases have presented with notable edema and peau d'orange skin changes [9, 30] consistent with inflammatory carcinoma. There are also reports of gigantomastia with bilateral axillary swelling [32] due to similar hypertrophic processes affecting accessory axillary tissue [9]. In some cases, axillary swelling occurred after mastectomy [29, 35]. The authors concluded that clinical and cytological presentation of gigantomastia in pregnancy could be misleading and that careful cytomorphological evaluation could avoid misdiagnosis of malignancy [9, 33, 44].

Although non-Hodgkin lymphoma is rare in pregnancy, there are a few reported cases of confirmed malignancy presenting as gestational gigantomastia. In 1999, Windom and McDuffie Jr. [45] described a case of a 26-year-old gravida 2, para 1, with a twin gestation, who presented at 28 weeks of gestation with rapidly enlarging breasts, diplopia, and jaw pain. Initial lab investigations were normal; however, upon further investigation, her breast enlargement was found to be due to infiltration with high-grade, small, noncleaved cell lymphoma, with involvement in the axilla, liver, bilateral kidneys, retroperitoneum, and meninges. Despite receiving aggressive chemotherapy and radiation, the patient died 9 months after diagnosis [45]. Underlying malignancy presenting as bilateral gigantomastia was also reported by Vandenberghe and colleagues in 2005 [46], in which biopsy of the upper anterior mediastinum and bilateral breast tissue confirmed a diagnosis of T-cell lymphoblastic lymphoma [46]. Non-Hodgkin lymphoma was also reported by Sherer et al. [47] in 2004, describing a woman presenting with breast swelling and bilateral axillary lymphadenopathy. Thorough workup is therefore required to either rule out malignancy or provide an early diagnosis so that patients are able to make informed decisions regarding their pregnancy before receiving treatment. Clinical findings with respective histology and response to treatment from previous cases of gestational gigantomastia are outlined in Table 2.

Considering the range of differential diagnoses for a woman presenting with gigantomastia in pregnancy, a thorough workup should include white blood cell count, hematocrit level, platelet count, electrolyte panel, hormone profile (estrogen, progesterone, and prolactin), and tissue biopsy [3]. Histologic features of gigantomastic tissue commonly include glandular hyperplasia, abundant stromal tissue, acinar/periacinar stromal fibrosis, interstitial edema, and, in some cases, lymphocytic infiltration [6, 9, 19, 48]. Furthermore, there have been reported cases of hypercalcemia with gigantomastia, possibly attributed to excessive production of PTHrP by hypertrophied breast tissue [50–52]. Thus, patients presenting with gigantomastia should also be worked up for hypercalcemia, since high levels may warrant bilateral mastectomy [53]. Deranged liver function tests have also been reported and should be explored to rule out concurrent liver disease [54].

Treatment for gestational gigantomastia has varied on a case-by-case basis. Conservative measures include proper brassiere support, good skin hygiene, analgesia, and adequate nutrition [39]. While conservative management in most cases has resulted in spontaneous resolution of tissue hypertrophy in the postpartum period [7, 39, 49], medical and surgical interventions are warranted in cases that fail to regress or present with the aforementioned complications.

To date, bromocriptine is the most widely used medical regimen for the treatment of gestational gigantomastia, but results have been variable. Anecdotal evidence has demonstrated that bromocriptine therapy during pregnancy may lead to fetal intrauterine growth retardation; therefore it is suggested that fetal growth be monitored if bromocriptine is used during the course of pregnancy. Case reports have demonstrated that using bromocriptine during pregnancy is useful in preventing and healing necrotic breast ulcerations, arresting breast enlargement, promoting regression, and decreasing the need for surgery [20]. Furthermore, bromocriptine may be continued during the postpartum period to suppress lactation and breast growth, allowing surgical intervention [3, 6, 7, 31, 42].

The most successful treatment for gestational gigantomastia is surgical intervention [37, 48, 55]. Although the exact risks of nonobstetric surgery to the fetus are debatable [56, 57], surgical intervention may be indicated in cases of massive hemorrhage, ulceration, sepsis, and breast necrosis [20, 29, 43]. Surgery during the immediate postpartum period may be indicated to prevent further complications such as puerperal mastitis, in cases of enormous hypertrophy in which reduction is unlikely with bromocriptine therapy alone [19, 41] or in cases of continued growth despite medical therapy [58]. Since there is a possibility of recurrence with simple mastectomy or reduction mammoplasty, bilateral mastectomy with reconstruction is the treatment of choice in women who desire future pregnancies [3, 12, 21, 27, 43, 48, 55, 59–61]. Another advantage of bilateral mastectomy over reduction or simple mastectomy is less blood loss during the procedure, which is significant considering patients with gestational gigantomastia have engorged and friable blood vessels [19, 58, 60].

## 4. Conclusion

Gestational gigantomastia is a psychological and physically debilitating disease of unknown etiology. In order to rule out underlying disease processes which can present as gigantomastia, a full workup including hematology, endocrinology, and biopsy should be completed. For cases in which prolactin is elevated or normal, bromocriptine therapy during the course of pregnancy has been used, yielding variable results. Fetal growth should be monitored with bromocriptine therapy due to case reports describing intrauterine growth retardation. Finally, the definitive treatment for gestational gigantomastia is surgical. Women who desire future pregnancies would benefit most from bilateral total mastectomy, since there is an increased risk of recurrence following simple mastectomy or reduction mammoplasty.

## 5. Summary of Recommendations

1. Immediate workup for a patient presenting with unilateral or bilateral gigantomastia includes
  - standard prenatal labs;
  - CBC with differential;
  - serum chemistry panel;
  - liver function tests;
  - serum calcium and albumin levels;
  - hormone profile: estrogen, progesterone, and prolactin.
2. Send the following tests for anti-dsDNA, ANA, RF, anti-Smith, CCP, antithyroglobulin, and anti-TPO, in addition to ESR and CRP, to investigate possible concomitant autoimmune disorders.
3. To evaluate malignancy, perform a breast biopsy. Any finding suspicious of malignancy should be followed up with an MRI of the head and CT scan of the thorax, abdomen, and pelvis to detect areas of metastasis.
4. Surgical intervention is not recommended in uncomplicated cases of gestational gigantomastia due to potential fetal compromise.
5. Delivery via cesarean section or induction of labor is recommended for pregnancies complicated by breast necrosis, maternal sepsis, or hemorrhage.
6. Trial of postpartum bromocriptine (2.5 mg twice daily) with cessation of breastfeeding is recommended to possibly reduce breast size before surgical intervention. If bromocriptine is used during pregnancy, serial fetal growth monitoring is recommended.
7. Final surgical intervention with bilateral total mastectomy instead of reduction mammoplasty or simple mastectomy is recommended, especially in women desiring future pregnancy.

## Figures and Tables

**Table 1:** Breast dimensions of patient with gestational gigantomastia on 22/7/2014, postpartum day 7.

	Right breast (cm)	Left breast (cm)
Breast circumference	69	65
Breast length from the base to tip of nipple	30	31
Breast length from underarm to tip of nipple	47	49
Total chest circumference (including the back and breasts)	134 cm.	

**Table 2:** Summary of gestational gigantomastia cases with normal prolactin levels.

Year	Clinical presentation	Unique laboratory/radiology findings	Sample: histology	Treatment and outcome
*Final diagnosis of benign gestational macromastia*				

1947 [2]	18-year-old with rapidly enlarging, tender, and ulcerated breasts in 3 sequential pregnancies, progressing to sepsis in first pregnancy.	Low urinary steroid metabolites (pregnanediol, estrogen, and 17-ketosteroids).	Biopsy of breast and axillary lymph node: chronic breast abscess, lactating breast, and hyperplastic lymph node.	Estrogen therapy: clinical worsening. Testosterone therapy: no improvement. Antibiotic therapy for ulceration and sepsis. Spontaneous reduction postpartum.

1954 [2]	20-year-old with bilateral breast enlargement and left breast pain, redness, swelling, and discharge in second pregnancy and unilateral nodular enlargement in third pregnancy.	Low urinary steroid metabolites (pregnanediol, estrogen, and 17-ketosteroids).	N/A	Norethindrone: no further growth during pregnancy with increased urinary estrogen excretion. Spontaneous reduction postpartum.

1965 [21]	22-year-old G2 with a history of myasthenia gravis, fibrocystic change, and idiopathic breast enlargement status—after bilateral reduction mammoplasty, with bilateral breast enlargement and skin thinning during the first trimester of pregnancy.	None.	Mastectomy tissue: fibroadenomatosis with extensive adenosis and proliferation of glandular elements, dilated ducts, and increased vascularization.	Bilateral subcutaneous mastectomy.

1986 [42]	32-year-old G5 at 21 weeks, with bilateral breast enlargement, skin ulceration, severe pain, and difficulty ambulating.	None.	Mammoplasty tissue: proliferation of intermediate-sized ducts with atypical lobules, variable stromal fibrosis, edema, and focal fat necrosis without atypia.	Bilateral reduction mammoplasty. Postpartum bromocriptine 2.5 mg twice daily × 14 days: no signs of breast enlargement.

1987 [32]	28-year-old G3 at 19 weeks with history of multiple bilateral fibroadenomas, with bilateral breast enlargement, local tenderness, backache, and bilateral axillary breast tissue.	None.	Mastectomy tissue: extensive collagenous stroma with atrophic lobules.	Symptomatic relief with bromocriptine (2.5 mg twice daily), partial regression with injectable medroxyprogesterone acetate. Eventual bilateral total mastectomy.

1988 [43]	34-year-old gravid woman at 8-week GA with history of fibroadenomas, with bilateral breast enlargement, erythema, localized numbness, enlarged veins, and eventual tissue necrosis.	None.	Mastectomy tissue: multiple fibroadenomas with hyperplasia and multiple lactating adenomas.	Reductive mastectomy: no recurrence with subsequent pregnancies.

1995 [41]	30-year-old G3 at 12 weeks with bilateral breast enlargement and limitation of daily activities, progressing to skin atrophy, infection, severe pain, and difficulty breathing.	None.	Fine-needle biopsy: cellular hypertrophy with no evidence of malignancy. Mammoplasty tissue: lobular hyperplasia, connective tissue edema, and distension of secretory ducts.	Refractory to tamoxifen and furosemide therapy. Antibiotics for skin infection. Eventual postpartum reduction mammoplasty with no recurrence.

2002 [31]	24-year-old G2 at 19 weeks with bilateral, rapidly enlarging breasts, pain, and difficulty with daily activities; with eventual ulceration.	None.	FNA: benign ductal cell hyperplasia with normal lobular structure.	Rest, dressings, antibiotics, progesterone, and furosemide: continued growth and edema. Elective cesarean at 34 weeks, bromocriptine therapy: reduction in breast size with eventual mammoplasty.

2003 [6]	24-year-old G2 with bilateral, rapidly enlarging breasts, pain, difficulty breathing and eventual skin atrophy, venous engorgement, ulceration, necrosis, and hemorrhage.	None (all labs within normal limits).	Mastectomy tissue: lobular hyperplasia, dilated ducts, pseudoangiomatous hyperplasia, interstitial edema, and increased fat and connective tissue.	Bilateral simple mastectomy: no regrowth.

2004 [13]	28-year-old with gradual, left-sided breast enlargement throughout pregnancy, presenting postpartum with persistent painful breast enlargement.	Fine-needle aspiration diagnosis of phyllodes tumor.	Mastectomy tissue: lactational changes, adenosis, and periductal and diffuse fibrosis. No features of phyllodes tumor or carcinoma.	Simple mastectomy, no recurrence at one-year follow-up.

2005 [12]	23-year-old with unilateral breast enlargement persisting beyond delivery.	Tumor markers: *increased CA19-9*. Ultrasound: homogenous mass with hypoechoic dilation of ducts. Immunohistochemistry stain: *CA19-9 positive*.	Mammoplasty tissue: abundant proliferation of stromal loose connective tissue interposed with fat cells surrounding normal and lactating lobules.	Elective tumorectomy and mammoplasty.

2007 [48]	23-year-old G2 at 12 weeks with bilateral breast enlargement, serous nipple discharge, hyperpigmentation, venous dilation, ulceration, and axillary lymphadenopathy.	Excisional biopsy: 20% epithelial cells with moderate ER and PR positivity.	Excisional biopsy of breast tissue and axillary lymph node: adenosis, moderate epitheliosis, fibrosis, and stromal B-lymphocytic infiltration.	Refractory to conservative management with NSAIDs, diuretics, and analgesics. Refractory to bromocriptine therapy. Eventual bilateral subcutaneous mastectomy with flap repair, without recurrence.

2008 [19]	30-year-old G2 with twin pregnancy and history of myasthenia gravis, with bilateral breast enlargement, mastalgia, back pain, difficulty breathing, decreased range of motion, and insomnia from onset of pregnancy.	None.	Mastectomy tissue: dense lobular units containing dilated acinar units filled with eosinophilic proteinaceous material, separated by loose connective tissue, with collagen-fiber stroma and lymphocytic infiltration.	Bilateral reduction mammoplasty followed by bromocriptine therapy.

2011 [7]	24-year-old G3 with bilateral breast swelling, milky discharge, pain, bilateral axillary lymphadenopathy, and limited range of motion of upper body.	Leukocytosis.	N/A	Conservative management with antibiotics, daily dressings and analgesics: spontaneous reduction postpartum.

2015 [37]	27-year-old at 30 weeks with a history of joint inflammation and morning stiffness before pregnancy, and bilateral breast enlargement, pain, and axillary breast tissue growth since early pregnancy.	Anemia of chronic disease, positive ANA titer, positive APA titer, and hypocomplementemia.	Biopsy of breast tissue: sclerosing adenosis with usual hyperplasia.	Refractory to bromocriptine therapy. Postpartum bilateral mastectomy with excision of accessory glands: no recurrence.

2015 [49]	32-year-old G5 at 33 weeks with history of bilateral breast enlargement in all previous pregnancies, with bilateral breast enlargement, back and neck discomfort, limitation of movement, erythematous skin, and dilated veins.	None.	Histology reported consistent with normal breast tissue.	Conservative management. Complete resolution postpartum.

*Final diagnosis of malignancy*				

1998 [45]	26-year-old G2 with twin gestation at 28 weeks, with bilateral, rapidly enlarging breasts, headache, diplopia, and jaw pain.	Elevated LDH. CT: nodal masses in left axilla, liver, bilateral kidneys, and retroperitoneum. MRI of the head with contrast: prominent meningeal enhancement with possible lymphomatous involvement.	Nodule in labium majus found during episiotomy repair: dermal infiltration with high-grade, *small, noncleaved cell lymphoma.*	Preterm induction of labor due to elevated LDH. Postpartum systemic chemotherapy, radiation therapy, intrathecal methotrexate, and bone marrow transplant: death 9 months after diagnosis.

2005 [46]	29-year-old with twin gestation at 33 weeks, bilateral breast enlargement, and dilated superficial veins. Postpartum development of shortness of breath.	Ultrasound: large hyporeflective nodes in both breasts with hypervascularization and fibrosis MRI: large solid-tissue mass in upper anterior mediastinum.	Biopsy of mediastinal mass and right breast: diffuse lymphoid infiltration. Immunohistochemical analysis: *T-cell lymphoblastic lymphoma.*	Cesarean delivery at 33.4 weeks due to preterm labor. Refractory to postpartum bromocriptine, cabergoline, and erythromycin. Follow-up with combination chemotherapy × 12 months. No further evidence of disease.
